# FogGate-YOLO: Traffic Object Detection in Foggy Environments Using Channel Selection Mechanisms

**DOI:** 10.3390/s26061811

**Published:** 2026-03-13

**Authors:** Yuhe Yang, Suilian You, Jinpeng Yu, Bo Lu

**Affiliations:** 1 School of Physics, Changchun University of Science and Technology, Changchun 130022, China; 2 Zhongshan Institute of Changchun University of Science and Technology, Shuma Building, Zhongshan 528437, China; 3 School of Optoelectronic Engineering, Changchun University of Science and Technology, Changchun 130022, China

**Keywords:** foggy environment, visual sensors, object dection, attention mechanism

## Abstract

To address the challenges posed by foggy conditions in object detection tasks, we propose FogGate-YOLO, an enhanced YOLOv8 framework designed for robust and efficient detection in foggy environments. Unlike traditional methods that rely on image dehazing or preprocessing enhancements, our approach directly strengthens the model’s feature representation by introducing two novel modules: GroupGatedConv and C2fGated. These modules collaboratively mitigate fog-induced degradation, improving feature extraction and enhancing performance without additional inference overhead. The GroupGatedConv module focuses on coarse-grained channel selection in the early to mid-stages of the backbone, suppressing noise while preserving essential structural features. The C2fGated module refines the aggregated features in both the backbone and neck after multi-branch fusion, enhancing fine-grained feature recalibration. Together, these two modules provide a hierarchical coarse to fine channel selection strategy that significantly improves the model’s discriminative power in foggy conditions.

## 1. Introduction

Object detection plays a vital role in the field of autonomous driving, as it directly impacts the accuracy and reliability of the system [1]. However, in foggy weather conditions, various challenges emerge, including reduced visibility and blurred object boundaries, which can significantly degrade the performance of detection algorithms [2]. These issues not only compromise the system’s effectiveness but also raise concerns about the safety and dependability of autonomous vehicles [3]. Therefore, researching object detection in foggy weather scenarios is crucial for enhancing the robustness and reliability of autonomous driving systems.

In recent years, notable advancements have been made in addressing the challenge of object detection under foggy weather conditions [4,5]. Traditional approaches predominantly rely on conventional computer vision techniques such as edge detection, filtering, and background modeling. While these methods offer partial solutions for foggy images, their performance in complex scenes and under severe fog conditions is limited. To improve object detection in such environments, researchers have increasingly turned to physical models for representing foggy images. For example, He et al. [6] proposed a single-image dehazing method based on the dark channel prior, and Zhu et al. [7] introduced a fast dehazing approach utilizing color attenuation priors. These dehazing techniques enhance the visibility of foggy images, which, in turn, boosts the accuracy of object detection. However, methods based on physical models typically require the estimation of fog density, which makes it challenging to handle varying fog densities across diverse and complex scenes.

With the continuous advancement of deep learning techniques, object detection has gradually become a major research focus [8,9], leading to the emergence of numerous high-performance algorithms based on convolutional neural networks (CNNs). Existing detection methods are generally categorized into two types: two-stage detectors and one-stage detectors. Two-stage detectors, represented by R-CNN [10], Fast R-CNN [11], and Faster R-CNN [12], typically generate a set of candidate regions and then perform classification and position regression for each proposal. For example, Faster R-CNN introduces a Region Proposal Network (RPN) to efficiently generate candidate boxes and employs ROI Pooling for feature extraction and regression, achieving strong detection accuracy. However, these region proposal-based approaches require considerable computational resources and often suffer from slower inference speeds, making them less suitable for real-time applications with strict latency constraints [13], such as autonomous driving. In foggy weather scenarios, Chen et al. further proposed a domain-adaptive detection method [14] that aligns features between source and target domains to improve performance under degraded visibility, highlighting the importance of robustness in adverse environments.

In contrast, one-stage detectors directly perform classification and localization on the input image without generating candidate boxes, significantly improving detection efficiency. Representative algorithms in this category include the YOLO series [15,16] and SSD, where YOLO divides the image into grids to predict bounding boxes and class probabilities, while SSD predicts multi-scale bounding boxes across different feature layers. Owing to their high speed and relatively simple architecture, one-stage detectors are widely adopted in real-time vision tasks. Nevertheless, improving accuracy while maintaining lightweight and efficient computation remains a critical challenge. To address this issue, many studies have explored structural optimization and feature enhancement strategies for YOLO-based models. For instance, Qiu et al. [17] enhanced YOLOv5 by integrating the Coordinated Attention (CA) mechanism [18] with GhostNet [19], improving feature representation capability in complex environments. Fan et al. [20] combined YOLOv5 with dark channel enhancement to alleviate low-illumination problems during nighttime image capture and compared multiple image enhancement methods to validate performance improvements. Baidya et al. [21] introduced an additional detection head and incorporated ConvMixer [22] modules for unmanned aerial vehicle detection tasks, demonstrating competitive performance on the VisDrone2021 dataset. Similarly, Ge et al. [23] embedded Coordinated Attention and Squeeze-and-Excitation (SE) modules [24] into the YOLOv5s architecture to strengthen channel feature learning, further enhancing detection performance. These studies indicate that although one-stage detectors provide significant advantages in speed, continuous improvements in attention mechanisms, feature fusion strategies, and lightweight network design are still required to achieve a better balance between accuracy and efficiency, especially under challenging conditions such as foggy weather and complex lighting environments.

Moreover, several studies have investigated the impact of heavy fog on vehicular sensors and the accuracy of object detection in driving environments. For example, Ogunrinde et al. [25] analyzed how the performance of CNN-based target detectors deteriorates rapidly under adverse weather conditions, and examined methods to defog and restore the quality of foggy images to improve real-time detection performance. Liu et al. [3] conducted a quantitative analysis on how varying visibility levels affect the accuracy of visual sensors in foggy conditions. Their findings showed that as fog density increased, the accuracy of object detection using Faster R-CNN decreased significantly, with detection accuracy dropping from 91.55% under clear weather to 57.75% under heavy fog.

Over the past two years, significant advancements have been made in object detection methods based on deep learning. Zhang et al. [26] improved YOLOv8 by adding a small-object-specific detection layer and a CEF module with CDW-EMA attention, enhancing multiscale perception and background suppression. Wang et al. [27] proposed MDD-ShipNet by integrating a CNN-based dehazing mechanism with a multi-scale feature fusion dynamic head, enhancing ship detection performance in foggy environments. Zhang et al. [28] proposed HR-YOLO, an improved YOLOv8 model for foggy ADAS detection of vehicles and pedestrians, incorporating EHPD-Net backbone, DND-Net defogging, optimized neck fusion, and WIoU loss, yielding mAP gains of 5.9% on RTTS and 9.7% on Foggy Cityscapes. Wang et al. [29] proposed YOLO-Extreme, an enhanced YOLOv12-based framework incorporating DBB, MCAM, and CSFB modules for robust obstacle detection in foggy environments, achieving 50.1% mAP on RTTS (3.9% higher than baseline) with real-time speed for visually impaired navigation assistance. Liu et al. [30] proposed an optimized YOLOv5-based target detection algorithm inspired by plant intelligence, integrating GCAnet for polarization image fusion and Wise_IOU loss with adaptive plant growth mechanisms, achieving 78.4% mAP in foggy conditions, outperforming existing models.

In the aforementioned research on foggy weather object detection, although the detection accuracy has been improved, most of these methods are primarily focused on defogging and image enhancement [31]. This study aims to enhance object detection performance in foggy environments by introducing a selective mechanism for intermediate-level feature representation, thereby strengthening the network’s ability to capture discriminative information and improving the robustness of foggy-scene target detection. In recent years, attention mechanisms have been widely applied in computer vision due to their strong capability in enhancing feature representation and modeling contextual relationships [24,32]. Inspired by these advances, this study innovatively proposes two gated modules based on attention mechanisms and integrates them into the YOLOv8 framework to improve road object detection performance under foggy weather conditions.

While our work draws inspiration from attention mechanisms, it differs fundamentally from traditional approaches. Conventional attention modules, such as SE and ECA, typically generate weights for feature recalibration based on input feature maps, lacking the capability for feature extraction or fusion. In contrast, our proposed GroupGatedConv and C2fGated modules innovatively integrate channel selection mechanisms into core operations: GroupGatedConv performs channel selection while conducting feature extraction, preserving the local feature capture capability of convolutions; whereas C2fGated introduces dynamic channel filtering based on the multi-scale feature fusion advantage of the C2f structure. This design enables the modules to adaptively enhance crucial channel features like attention mechanisms, without sacrificing their original feature processing capabilities, thereby significantly improving the model’s representation ability.

The main contributions of this study are as follows:

1. We propose the GroupGatedConv module, which uses group-wise gating to improve feature selection in foggy conditions while maintaining computational efficiency;
2. Based on the C2f module, we propose a new module, “C2fGated”, which embeds the ECA attention mechanism [33] before feature fusion and after the final convolution operation to enhance the selection of target channel features;
3. We integrate the “C2fGated” and “GroupGatedConv” modules into YOLOv8 to enhance the intermediate feature selection capability, thereby improving its ability to detect road targets in foggy conditions.

The structure of this paper is organized as follows. Section 2 provides an overview of the original YOLOv8n model and discusses the innovations introduced in this study. In Section 3, we present the dataset, experimental setup, and the results of our experiments. Finally, Section 4 concludes the paper and outlines potential directions for future research.

## 2. FogGate-YOLO

### 2.1. Overview of YOLOv8

YOLOv8 [34], released by Ultralytics in January 2023, is the latest evolution in the YOLO series, emphasizing real-time performance, accuracy, and ease of use. Its architecture comprises three main parts: a backbone based on CSPDarknet [35] with C2f modules (replacing the C3 module from prior versions) that stack bottleneck layers, concatenate outputs for richer gradient flow, and enable efficient multi-scale feature extraction while keeping the model lightweight. The neck uses a PAN-FPN structure [36] for bidirectional feature fusion, combining top-down semantic information with bottom-up spatial details to handle objects of varying scales effectively. The head adopts an anchor-free design with decoupled branches for classification and regression, employing Distribution Focal Loss (DFL) [37] and Complete IoU (CIoU) [38] to improve bounding box alignment, objectness, and localization without relying on predefined anchors.

These structural features provide key task advantages: superior handling of multi-scale and small objects in complex scenes, faster post-processing due to the anchor-free approach, and excellent balance between speed and precision. YOLOv8 supports scalable variants (nano to extra-large) for diverse hardware, and extends to tasks like instance segmentation, pose estimation, and classification, making it highly versatile for real-time applications in surveillance, autonomous systems, and industrial monitoring. Considering the real-time detection requirements in foggy weather conditions, this study employs YOLOv8n as the experimental model.

### 2.2. The Architecture of FogGate-YOLO Network

**Figure 1:**
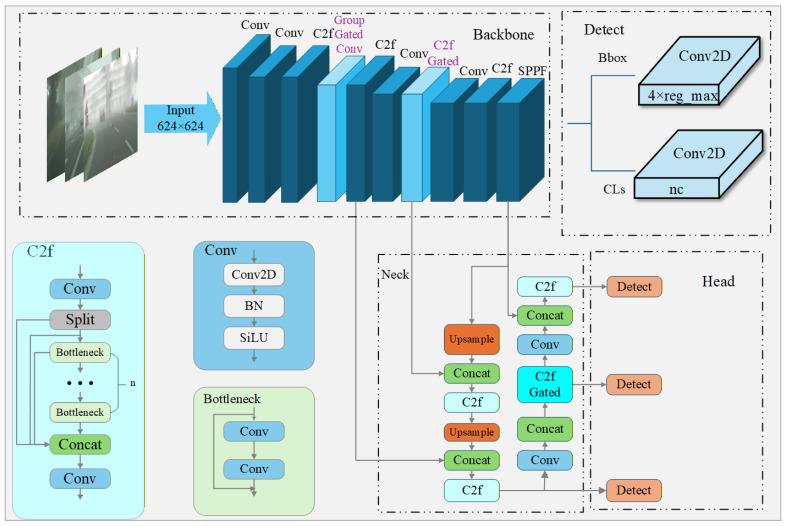
The network architecture of FogGate-YOLO introduces two self-designed modules, GroupGatedConv and C2fGated, to enhance the model’s feature selection ability, improving its performance in foggy conditions.The purple text in the figure represents the changes made to the Backbone, while the light blue text represents the changes made to the Neck.

In foggy weather conditions, the scattering of atmospheric particles can significantly degrade images, leading to reduced contrast, altered colors, and making it challenging to identify object features [39]. Therefore, selectively attending to channel-wise features of objects in foggy conditions becomes critically important for enhancing detection performance under such adverse weather. In this paper, we propose a novel approach to address the aforementioned issue by improving the YOLOv8n network architecture. Our proposed network, FogGate-YOLO, is illustrated in Figure 1.

Traditional convolution modules primarily focus on generating feature maps without an explicit mechanism for feature selection. However, in foggy conditions, object detection differs significantly from clear-weather scenarios, as fog degrades spatial details while channel-wise feature maps provide more discriminative cues for algorithms to identify targets. To address this, we propose the Channel Group Gated Convolution (GroupGatedConv) module, which introduces group-wise gating to selectively suppress or retain channel groups, enhancing channel-specific feature representation critical for foggy target detection.

Moreover, traditional C2f modules primarily focus on feature fusion through splitting, bottleneck stacking, and concatenation, but lack an explicit mechanism for channel-wise feature selection after fusion. In foggy conditions, spatial details are severely degraded by haze, while channel-level cues remain more discriminative for target identification. To address this limitation, we propose the C2fGated module, which integrates the Efficient Channel Attention (ECA) mechanism immediately after the concatenation step. As introduced in ECA-Net, ECA computes an adaptive kernel size and directly applies a 1D convolution on the channel dimension, enabling each channel to learn its relative importance with respect to its neighboring channels without dimensionality reduction. This design allows the module to perform targeted channel selection on the fused mid-level features, significantly improving robustness for small-target detection under heavy fog.

### 2.3. Construction of Object Detection Model for Foggy Scenes

#### 2.3.1. The GroupGatedConv Module

The design of the GroupGatedConv module draws inspiration from channel attention mechanisms such as Squeeze-and-Excitation (SE) [24] and Efficient Channel Attention (ECA), which emphasize selective channel weighting to improve feature discriminability while minimizing computational overhead. To reduce complexity further, we adopt a group-gated convolution approach, partitioning output channels into groups. This enables efficient channel selection without the full pairwise dependencies of standard attention, making it suitable for real-time applications like object detection in road target detection.

The module processes an input feature map $x\in R^{B\times c_{1}\times H\times W}$ through two parallel branches. In the main convolution branch, features are extracted as follows:

(1) $$y=\sigma (\mathrm{BN}\left({\mathrm{Conv}}_{k\times k,s,p}\left(x\right)\right)),$$

where ${\mathrm{Conv}}_{k\times k,s,p}$ is a standard 2D convolution with kernel size *k*, stride *s*, and padding *p* (typically k=3, s=1, p=1), BN denotes batch normalization, and σ is the SiLU activation function, yielding $y\in R^{B\times c_{2}\times H^{\prime }\times W^{\prime }}$.

In the gating branch, global information is compressed via adaptive average pooling:

(2) $$p=\mathrm{AdaptiveAvgPool}2d\left(y\right)\in R^{B\times c_{2}\times 1\times 1}.$$

A 1×1 convolution followed by sigmoid activation maps this to group-wise gates, ensuring $c_{2}$ is divisible by the number of groups *G* (e.g., G=8):

(3) $$g=\mathrm{Sigmoid}\left({\mathrm{Conv}}_{1\times 1}\left(p\right)\right)\in R^{B\times G\times 1\times 1}.$$

To apply group gating, the output *y* is reshaped into groups with channel group size $c_{g}=c_{2}/G$:

(4) $$y^{\prime }=\mathrm{Reshape}\left(y\right)\in R^{B\times G\times c_{g}\times H^{\prime }\times W^{\prime }}.$$

The gates are expanded and multiplied group-wise:

(5) $$o^{\prime }=y^{\prime }⊙g.\mathrm{unsqueeze}\left(2\right),$$

followed by reshaping back to the original dimensions:

(6) $$o=\mathrm{Reshape}\left(o^{\prime }\right)\in R^{B\times c_{2}\times H^{\prime }\times W^{\prime }}.$$

**Figure 2:**
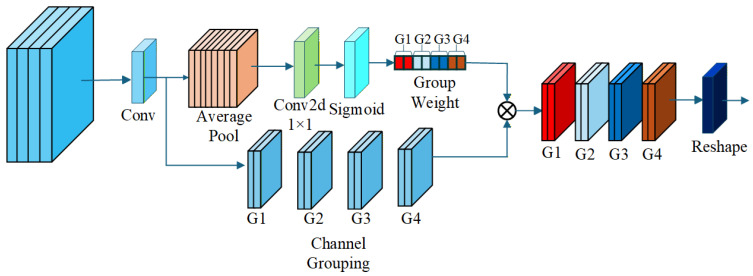
Our GroupGatedConv uses gated convolutions for feature selection while reducing overhead. We integrate it into the fourth layer of the YOLOv8 backbone for efficient feature extraction.ifferent colors represent feature maps from different groups, circles indicate multiplication operations, and arrows represent the flow direction of feature maps.

Given that mid-level features play a dominant role in distinguishing foggy targets, where channel cues outweigh degraded spatial details, we integrate the GroupGatedConv module into the fourth layer of the YOLOv8 backbone. This placement allows selective channel enhancement at a stage where features balance locality and abstraction, optimizing detection in fog without excessive overhead. Integrating into shallow layers could disrupt low-level texture and edge extraction, as gating could prematurely suppress primitive features essential for initial representation. Conversely, deep-layer integration risks interfering with high-level semantic aggregation, where overly aggressive gating could diminish object wholeness and contextual understanding critical for final predictions. The architecture of the GroupGatedConv module is illustrated in Figure 2, as shown in the following diagram.

#### 2.3.2. ECA Attention Module

The Efficient Channel Attention (ECA) module, as introduced in the ECA-Net framework, represents a lightweight yet powerful channel attention mechanism designed to enhance the performance of deep convolutional neural networks (CNNs) while maintaining minimal computational overhead. Drawing from the analysis of SENet, ECA addresses key limitations in traditional channel attention, such as dimensionality reduction that can lead to information loss and excessive model complexity from fully connected layers. Its functional characteristics include: (1) avoiding dimensionality reduction to preserve channel-wise dependencies; (2) employing efficient local cross-channel interactions via 1D convolution, which captures dependencies with fewer parameters; and (3) adaptive kernel size selection based on channel dimensions, ensuring scalability across network depths. These advantages enable ECA to achieve significant performance, gains, e.g., over 2% Top-1 accuracy boost on ResNet-50 [40], with negligible increases in parameters and FLOPs, as demonstrated in image classification, object detection, and segmentation tasks.

The ECA module is engineered for efficient channel-wise feature recalibration, utilizing global average pooling followed by a parameter-efficient 1D convolution to model inter-channel relationships. The kernel size *k* is adaptively determined based on the input channel dimension $c_{1}$, using the formula:

(7) $$t=\left\lfloor \left|\frac{\log_{2}\left(c_{1}\right)+b}{\gamma }\right|\right\rfloor ,\;k=t\;\mathrm{if}\;t\;\mathrm{mod}\;2\neq 0\;\mathrm{else}\;t+1,$$

where γ=2 and b=1 are hyperparameters ensuring *k* is odd for symmetric padding.

The forward pass begins with global average pooling to compress spatial dimensions:

(8) $$y=\mathrm{AdaptiveAvgPool}2d\left(x\right)\in R^{B\times c_{1}\times 1\times 1}.$$

Next, the tensor is reshaped for 1D convolution:

(9) $$y^{\prime }=y.squeeze\left(-1\right).permute\left(0,2,1\right)\in R^{B\times 1\times c_{1}},$$

followed by the 1D convolution to capture local cross-channel interactions:

(10) $$y^{\prime \prime }=\mathrm{Conv}1d\left(y^{\prime }\right)\in R^{B\times 1\times c_{1}},$$

with kernel size *k* and padding k//2. Sigmoid activation generates the attention weights:

(11) $$w=\mathrm{Sigmoid}\left(y^{\prime \prime }\right)\in R^{B\times 1\times c_{1}}.$$

Finally, the weights are reshaped and broadcasted:

(12) $$w^{\prime }=w.permute\left(0,2,1\right).unsqueeze\left(-1\right)\in R^{B\times c_{1}\times 1\times 1},$$

and multiplied with the input:

(13) $$o=x⊙w^{\prime }.expand\_as\left(x\right).$$

**Figure 3:**
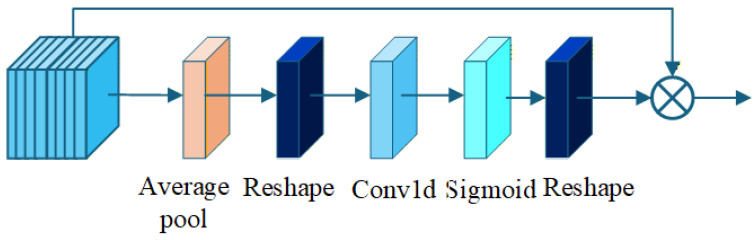
The Efficient Channel Attention (ECA) module is a lightweight channel attention mechanism that enhances CNN performance while minimizing computational overhead.Arrows represent the flow direction of feature maps, while circles indicate multiplication operations applied to the feature maps.

The architecture of the ECA module is illustrated in Figure 3, as shown in the following diagram.

#### 2.3.3. The C2fGated Module

The C2fGated module follows the standard C2f architecture with a lightweight channel attention enhancement. First, the input feature map is passed through a 1 × 1 convolution (cv1) and split into two equal channel groups. One group proceeds directly, while the other is fed into a stack of Bottleneck modules (controlled by hyper-parameter n). All branches are then concatenated along the channel dimension. At this point, the ECA module is applied to the concatenated feature, where it adaptively determines the convolution kernel size based on the total channel count and performs efficient local cross-channel interaction. Finally, a 1 × 1 convolution (cv2) is used to adjust the output channel dimension to c2. The entire process maintains the original C2f structure while adding negligible computational overhead.

**Figure 4:**
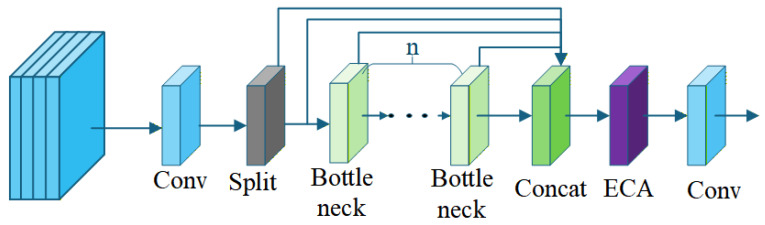
C2fGated enhances the C2f module by adding Efficient Channel Attention (ECA) for better channel-level feature selection. It improves small-target detection in foggy conditions by adaptively recalibrating channel importance, maintaining the original structure with minimal computational cost.

To achieve optimal mid-level feature selection for foggy target detection, the C2fGated module is embedded at the seventh layer of the YOLOv8 backbone and the nineteenth layer of the Neck. These positions correspond to stages where features possess a balanced combination of spatial details and semantic information, allowing ECA to most effectively recalibrate channel importance without disrupting low-level texture extraction in shallower layers or high-level semantic aggregation in deeper layers. The architecture of the C2fGated module is illustrated in Figure 4, as shown in the following diagram.

## 3. Experiments

This section presents a clear and concise summary of the experimental results, along with their interpretation and the conclusions drawn from the experiments.

### 3.1. Datasets

Adverse weather conditions, particularly dense fog, pose considerable challenges to object detection models based on convolutional neural networks (CNNs). Fog significantly degrades image quality by reducing contrast, blurring object edges, and obscuring critical visual features through atmospheric scattering, which often leads to increased false negatives and localization errors. To rigorously evaluate the true performance and robustness of our model under such demanding conditions, we employed a high-quality foggy weather dataset sourced from Roboflow, containing a total of 2975 images [41]. And this dataset consists of images captured under real fog conditions in actual environmental settings, offering a more authentic challenge for detection tasks. The dataset’s specialized focus on real-world fog scenarios makes it highly representative, providing a more accurate reflection of the model’s intrinsic detection capability in real fog conditions. Although the dataset is relatively moderate in size, its specialized focus on authentic and diverse foggy scenarios makes it highly representative and challenging, thereby providing a more accurate reflection of the model’s intrinsic detection capability rather than relying on large-scale data volume. We allocated 80% of the images (2380 images) as the training set and the remaining 20% (595 images) as the validation set to train and assess the model’s effectiveness, respectively.

### 3.2. Experimental Details

This research work used the PyTorch framework to complete a series of tasks using GPUs for accelerated training, and the specific relevant environment configurations are seen in Table 1.

Our algorithm was improved from YOLOv8n, with the learning rate set to 0.001 in the training phase and the weight decay value set to 0.0005. To optimize the parameters of the model, we used the stochastic gradient descent algorithm and the momentum optimization algorithm, and the input image had a length and width of 640 and a batch size of 32; the epoch of iteration was 200, and the momentum factor was 0.937. During the training process of this study, we employed various effective data augmentation techniques, including Mosaic, Hsv, Flip.

### 3.3. Evaluation Metrics

In foggy scenarios, object detection becomes particularly challenging due to visibility 296 degradation caused by atmospheric scattering, which often leads to missed detections. To 297 quantitatively evaluate the detection performance under such conditions, Recall and mean 298 Average Precision (mAP) are adopted as the primary evaluation metrics.

Among them, Recall is regarded as the most critical metric, as it directly reflects the model’s ability to detect existing targets in low-visibility environments. mAP is additionally reported to provide a comprehensive evaluation by jointly considering both detection accuracy and recall across different categories. Precision and Recall are defined as follows:

(14) $$\mathrm{Precision}=\frac{TP}{TP+FP}$$

(15) $$\mathrm{Recall}=\frac{TP}{TP+FN}$$

where TP denotes the number of true positive detections, FP represents the number of false positives, and FN indicates the number of false negatives.

In foggy scenes, a high Recall implies a lower missed-detection rate, which is particularly important for safety-critical applications such as autonomous driving.

The Average Precision (AP) for a single class is computed as the area under the Precision–Recall (P–R) curve:

(16) $$AP=\int_{0}^{1}P\left(R\right)\;dR$$

where P(R) denotes the precision as a function of recall.

The mean Average Precision (mAP) is obtained by averaging the AP values over all object categories:

(17) $$mAP=\frac{1}{N}\sum_{i=1}^{N}AP_{i}$$

where N is the total number of object classes and $AP_{i}$ denotes the average precision of the i-th class.

### 3.4. Comparison Experiments

**Figure 5:**
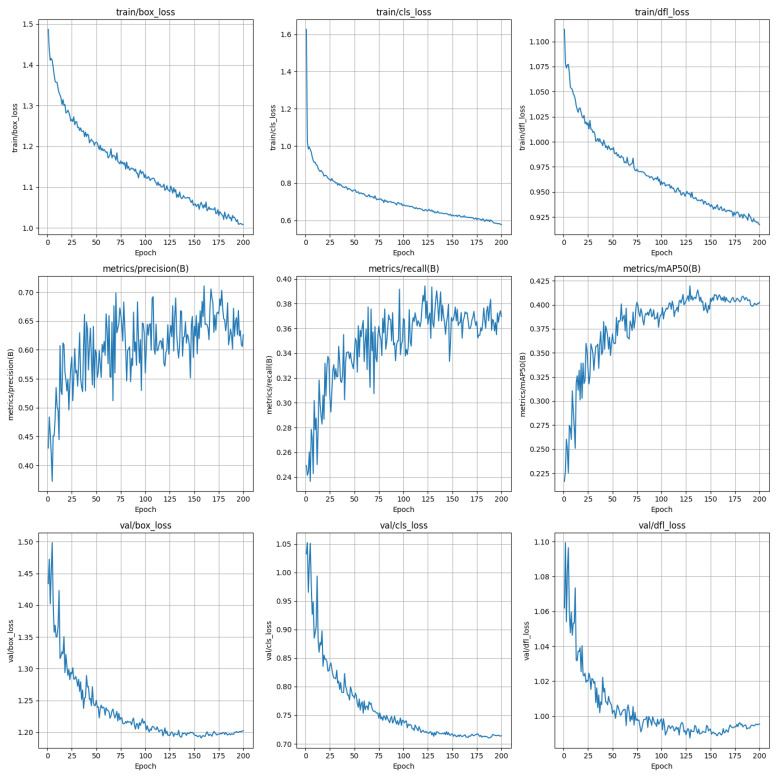
The visualization of various metrics during the training process, including bounding box loss, distribution focal Loss, class loss, Precision, Recall, mAP.

FogGate-YOLO provides an effective solution for object detection in foggy environments, aiming to address the severe impact of fog on detection accuracy. In traditional methods, image dehazing or enhancement is typically used as a preprocessing step. However, FogGate-YOLO directly strengthens the model’s feature extraction ability by introducing two novel modules, GroupGatedConv and C2fGated, which effectively mitigate the image degradation caused by fog. The GroupGatedConv module focuses on coarse-grained channel selection to suppress noise while preserving essential structural features, while the C2fGated module further refines the features post multi-branch fusion, enhancing the model’s discriminative power in foggy conditions. This design, based on modular enhancement, avoids the complexity of traditional image preprocessing and improves computational efficiency. Figure 5 illustrates the variations in key metrics, including bounding box loss, distribution focal Loss, and class loss, as well as Precision, Recall, mAP50 after each epoch during the training and validation process of FogGate-YOLO.

Compared to other enhanced YOLO models, FogGate-YOLO significantly improves detection accuracy in foggy environments while maintaining efficient inference. Experimental results show that FogGate-YOLO outperforms than models such as YOLOv5n and YOLOv8n in mAP50 and mAP50–95 metrics. FogGate-YOLO achieves an mAP50 of 41.3%, which is higher than other models in the YOLO series, such as YOLOv5n (39.8%), YOLOv6n (36.3%), YOLOv8n (40.6%), and YOLOv11n (39.6%). Meanwhile, FogGate-YOLO’s GFLOPs (8.8) are not significantly different from these models, with YOLOv8n having a GFLOPs of 8.8, YOLOv11n being 6.5, YOLOv6n at 11.4, and YOLOv5n at 7.7. This indicates that the two modules, C2fGated and GroupGatedConv, added to FogGate-YOLO do not introduce significant computational overhead, yet provide substantial performance improvements. Although FogGate-YOLO’s mAP50 (41.3%) is slightly lower compared to YOLOv5s (42.2%) and YOLOv8s (42.6%), FogGate-YOLO outperforms in terms of GFLOPs and parameter size. YOLOv5s has a GFLOPs of 24.0, YOLOv8s is 28.6, both significantly higher than FogGate-YOLO’s 8.8, and the parameter size for YOLOv5s is 9.1 M, for YOLOv8s it is 11.2 M, whereas FogGate-YOLO only has 3.152 M parameters. This demonstrates that while YOLOv5s and YOLOv8s have slightly better accuracy, they come with much larger computational demands and model sizes compared to FogGate-YOLO. Additionally, FogGate-YOLO’s Recall is 39.8%, which is higher than YOLOv5s at 37.8% and YOLOv8s at 39.3%. This indicates that FogGate-YOLO still performs exceptionally well in terms of target detection under foggy conditions, maintaining a high recall rate while keeping low computational overhead and small model size.

In summary, FogGate-YOLO demonstrates a balanced design, providing excellent performance in target detection under foggy conditions while maintaining computational efficiency and model lightweight, making it highly practical for real-world applications. The detection results of various algorithms on the dataset are shown in Table 2.

### 3.5. Ablation Experiments

To assess the impact of each module on the performance of our model, we performed an ablation study on our dataset. In order to maintain scientific rigor and ensure a thorough evaluation of the proposed model, we used three key metrics: Recall, mAP50, and mAP50-95. The influence of each individual module on the detection performance is summarized in Table 3.

### 3.6. The Impact of the GroupGatedConv Module

To further enhance channel-wise feature selection under foggy conditions while maintaining computational efficiency, we propose a lightweight GroupGatedConv module and evaluate its effectiveness through ablation experiments. Unlike conventional channel attention mechanisms that assign an individual weight to each output channel, the proposed GroupGatedConv adopts a group-wise gating strategy. Specifically, the output channels are evenly divided into multiple groups, and a single adaptive gate is learned for each group. This design enables the network to selectively suppress or preserve groups of feature channels according to their task relevance, while significantly reducing parameter count and computational overhead.

To assess the impact of early-stage channel selection, the proposed GroupGatedConv replaces the original convolution module at the fourth layer of the YOLOv8 backbone. This stage corresponds to mid-level feature extraction, where basic semantic patterns emerge while spatial resolution remains relatively high. By integrating GroupGatedConv at this layer, the network gains stronger selectivity over intermediate channel representations, enabling it to suppress fog-induced background responses and retain features that are more relevant to object structures. As a result, more informative and noise-resilient features are propagated to subsequent layers, improving the overall detection robustness under foggy conditions.

Compared with the baseline YOLOv8n, introducing the GroupGatedConv module leads to a Recall improvement of 0.8%. This gain indicates enhanced sensitivity to fog-obscured targets and demonstrates the effectiveness of group-wise channel gating for improving detection performance in degraded visual environments. Overall, the ablation results confirm that the proposed GroupGatedConv module strengthens mid-level feature discrimination with minimal computational overhead, making it a practical and effective component for foggy scene object detection.

### 3.7. The Impact of the C2fGated Module

By incorporating an Efficient Channel Attention (ECA) mechanism after feature concatenation, the proposed C2fGated module enables adaptive channel reweighting on the fused features. The ECA module captures local cross-channel dependencies through lightweight one-dimensional convolution, allowing the network to selectively emphasize task-relevant channels while suppressing noise-sensitive ones, without introducing dimensionality reduction or significant computational overhead.

To further investigate the influence of module placement, C2fGated is inserted into two critical stages of YOLOv8: the seventh layer of the backbone and the nineteenth layer of the neck. At the backbone stage, mid-level features begin to encode object structure and semantic cues while remaining susceptible to fog-induced interference. Introducing C2fGated at this stage facilitates channel-wise purification of intermediate representations, enhancing the stability of discriminative features propagated to deeper layers. At the neck stage, multi-scale features are fused to support detection at different resolutions. However, under heavy fog, small-target cues are often diluted during the fusion process. Embedding C2fGated after feature fusion allows the model to perform targeted channel selection on the fused mid-level features, significantly improving robustness for small-target detection in foggy scenes.

Compared with the baseline YOLOv8n, the proposed FogGate-YOLO achieves consistent performance improvements after introducing the single C2fGated module. Specifically, Recall is improved by 0.6%, mAP_50_ increases by 1.0%, and mAP_50–95_ improves by 0.6%. These results demonstrate that the proposed C2fGated module effectively enhances feature discrimination under foggy conditions and that the chosen insertion positions in both the backbone and neck contribute to the observed performance gains.

### 3.8. Joint Effect of C2fGated and GroupGatedConv

To further analyze the collaborative effect of the proposed modules, we conduct joint ablation experiments by simultaneously integrating C2fGated and GroupGatedConv into the YOLOv8n architecture. This setting allows us to evaluate whether the two channel selection mechanisms provide complementary benefits under foggy conditions.

The GroupGatedConv module is responsible for coarse-grained channel selection at early-to-mid stages of the backbone by suppressing or enhancing groups of correlated feature channels. This operation effectively filters out fog-induced background responses and preserves structurally meaningful features at the intermediate representation level. In contrast, the C2fGated module focuses on fine-grained channel recalibration after multi-branch feature fusion, enabling adaptive refinement of aggregated mid-level features in both the backbone and neck. When combined, these two modules form a hierarchical channel selection strategy. GroupGatedConv first performs structured channel filtering on mid-level features, providing a cleaner and more stable feature basis. Subsequently, C2fGated further reweights the fused features through efficient channel attention, selectively emphasizing task-relevant responses. This coarse-to-fine collaboration significantly strengthens the model’s ability to discriminate informative channels under moderate and heavy fog conditions.

**Figure 6:**
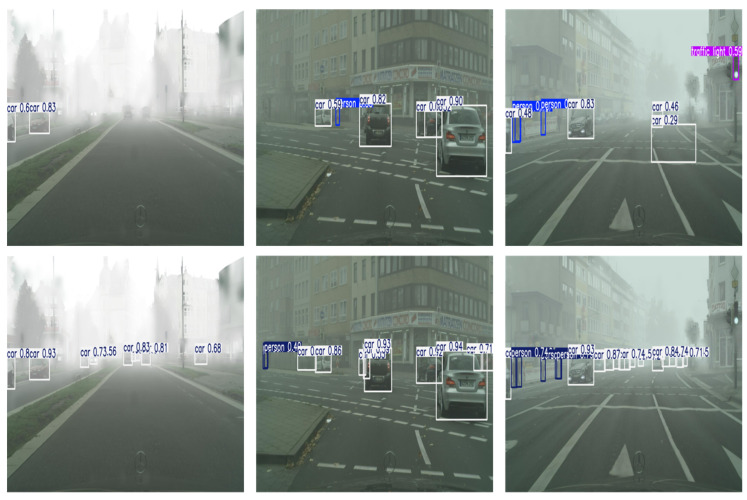
Partial detection results of YOLOv8n and FogGate-YOLO on the foggy dataset are shown above. The first row corresponds to YOLOv8n, the second row corresponds to FogGate-YOLO.

As a result, the proposed FogGate-YOLO exhibits a substantially enhanced capability for mid-level channel selection, which is particularly beneficial for road target detection in degraded visual environments. Compared with the baseline YOLOv8n, FogGate-YOLO achieves a Recall improvement of 2.6%, along with gains of 0.7% in mAP_50_ and 0.4% in mAP_50–95_. These results demonstrate that the cooperative integration of C2fGated and GroupGatedConv yields complementary and cumulative performance improvements, confirming the effectiveness of the proposed channel selection strategy for foggy scene object detection. With YOLOv8n’s GFLOPs at 8.8 and FogGate-YOLO’s GFLOPs also at 8.8, alongside YOLOv8n’s 3.151M parameters compared to FogGate-YOLO’s 3.152M parameters, this indicates that the two new modules introduce minimal computational overhead, further emphasizing the lightweight nature of FogGate-YOLO. It is evident from Figure 6 that FogGate-YOLO exhibits excellent detection capabilities in foggy weather conditions environments. Additionally, FogGate-YOLO can identify smaller objects in dense fog more effectively.

## 4. Conclusions

In this paper, we propose FogGate-YOLO, an enhanced YOLOv8 framework designed for robust object detection in foggy conditions. Unlike conventional approaches that rely on image dehazing or preprocessing enhancement, our method directly strengthens the model’s feature representation by embedding advanced channel selection mechanisms, effectively mitigating fog-induced degradation without additional inference overhead.

The core contributions are two synergistic modules: GroupGatedConv and C2fGated. GroupGatedConv performs coarse-grained channel selection in the early-to-mid backbone stages, suppressing fog-related noise while preserving structural features. C2fGated enables fine-grained recalibration after multi-branch fusion, refining aggregated features in both backbone and neck. Together, they form a hierarchical coarse-to-fine channel selection strategy that significantly boosts discriminative power under foggy scenes.

Extensive experiments demonstrate the effectiveness of FogGate-YOLO. Compared to the baseline YOLOv8n, it achieves improvements of 2.6% in Recall, 0.7% in mAP50, and 0.5% in mAP50–95 on foggy road detection datasets, with more pronounced gains under moderate and heavy fog. Joint ablation studies confirm the complementary benefits of the two modules. GroupGatedConv provides structured filtering at the mid-level, while C2fGated performs adaptive refinement after fusion. This coarse-to-fine collaboration creates a cleaner feature basis and selectively emphasizes task-relevant responses, markedly enhancing robustness in challenging foggy environments.

The results indicate strong potential for adverse weather detection. Although our algorithm has shown promising results, certain limitations remain. Firstly, the dataset used in the study has a relatively small sample size and lacks diverse scenarios, which restricts the model’s generalization ability. Secondly, key hyperparameters, such as the parameter G in the GroupGatedConv module, have not been explored in depth, and their impact on the model’s performance has not been thoroughly analyzed. Additionally, our algorithm has not been compared with the most advanced lightweight fog detection models in recent years, which represents a significant area for future research. In order to further enhance the performance of the algorithm, we plan to extend its application to various foggy scenarios in future work, analyzing its performance across different conditions. Additionally, we will apply other techniques such as data augmentation, transfer learning, and regularization to further improve the model’s generalization ability across different datasets. At the same time, we will conduct a detailed exploration of the hyperparameter G in the GroupGatedConv module, focusing on its impact across varying numbers of feature maps, especially in different network architectures. This will ensure that the algorithm can self-adaptively select the optimal number of groups, G, depending on the feature map quantity at different stages, thereby maximizing the model’s performance.

## Figures and Tables

**Table 1:** Experimental environment configuration.

Types	Environment
Operating System	Linux 5.15.0-113-generic
GPU	NVIDIA GeForce RTX 3090
CPU	AMDEPYC7402 24-Core Processor
Pytorch Version	2.5.0 + cu121
CUDA	12.1

**Table 2:** Detection results of different object detection methods.

Methods	Recall (%)	mAP50 (%)	mAP50–95 (%)	GFLOPs	Param (M)
YOLOv5n	35.2	39.8	23.6	7.7	2.6
YOLOv6n	32.4	36.3	22.4	11.4	4.7
YOLOv8n	37.2	40.6	24.9	8.8	3.151
YOLOv11n	35.2	39.6	24.3	6.5	2.6
YOLOv12n	35.0	39.6	24.0	2.6	6.5
YOLOv5s	37.8	42.2	26.4	24.0	9.1
YOLOv8s	39.3	42.6	27.1	28.6	11.2
FogGate-YOLO	39.8	41.3	25.4	8.8	3.152

**Table 3:** The impact of incorporating the component on the Precision and Recall of the model was evaluated on the foggy dataset. Among the variants, FogGate_1 represents the combination of YOLOv8n and C2fGated. FogGate_2 represents YOLOv8n and GroupGatedConv. Lastly, FogGate_3 combines YOLOv8n, GroupGatedConv, and C2fGated.

Methods	Recall (%)	mAP50 (%)	mAP50-95 (%)	GFLOPs	Param (M)
YOLOv8n	37.2	40.6	24.9	8.8	3.151
FogGate_1	37.8	41.6	25.5	8.8	3.151
FogGate_2	38.0	40.2	24.4	8.8	3.151
FogGate_3	39.8	41.3	25.4	8.8	3.152

## Data Availability

Data are contained within the article.

## References

[1] Bijelic M., Gruber T., Mannan F., Kraus F., Ritter W., Dietmayer K., Heide F. Seeing through fog without seeing fog: Deep multimodal sensor fusion in unseen adverse weather. Proceedings of the IEEE/CVF Conference on Computer Vision and Pattern Recognition.

[2] Walambe R., Marathe A., Kotecha K., Ghinea G. (2021). Lightweight object detection ensemble framework for autonomous vehicles in challenging weather conditions. Comput. Intell. Neurosci..

[3] Liu Z., He Y., Wang C., Song R. (2020). Analysis of the influence of foggy weather environment on the detection effect of machine vision obstacles. Sensors.

[4] Hahner M., Sakaridis C., Dai D., Van Gool L. (2021). Fog simulation on real LiDAR point clouds for 3D object detection in adverse weather. Proceedings of the IEEE/CVF International Conference on Computer Vision.

[5] Krišto M., Ivasic-Kos M., Pobar M. (2020). Thermal object detection in difficult weather conditions using YOLO. IEEE Access.

[6] He K., Sun J., Tang X. (2010). Single image haze removal using dark channel prior. IEEE Trans. Pattern Anal. Mach. Intell..

[7] Zhu Q., Mai J., Shao L. (2015). A fast single image haze removal algorithm using color attenuation prior. IEEE Trans. Image Process..

[8] Girshick R., Donahue J., Darrell T., Malik J. (2014). Rich feature hierarchies for accurate object detection and semantic segmentation. Proceedings of the IEEE Conference on Computer Vision and Pattern Recognition.

[9] Lin T., Goyal P., Girshick R., He K., Dollár P. Focal loss for dense object detection. Proceedings of the IEEE International Conference on Computer Vision.

[10] Liu W., Anguelov D., Erhan D., Szegedy C., Reed S., Fu C.Y., Berg C. SSD: Single Shot Multibox Detector. Proceedings of the Computer Vision–ECCV 2016: 14th European Conference.

[11] Girshick R. Fast R-CNN: Fast Region-based Convolutional Networks for Object Detection. Proceedings of the IEEE International Conference on Computer Vision (ICCV).

[12] Ren S., He K., Girshick R., Sun J. (2016). Faster R-CNN: Towards real-time object detection with region proposal networks. IEEE Trans. Pattern Anal. Mach. Intell..

[13] Yao J., Fan X., Li B., Qin W. (2022). Adverse Weather Target Detection Algorithm Based on Adaptive Color Levels and Improved YOLOv5. Sensors.

[14] Chen Y., Li W., Sakaridis C., Dai D., Van Gool L. Domain adaptive faster R-CNN for object detection in the wild. Proceedings of the IEEE Conference on Computer Vision and Pattern Recognition.

[15] Redmon J., Divvala S., Girshick R., Farhadi A. You Only Look Once: Unified, Real-Time Object Detection. Proceedings of the IEEE Conference on Computer Vision and Pattern Recognition.

[16] Jocher G., Stoken A., Borovec J., Chaurasia A., Changyu L., Hogan A., Hajek J., Diaconu L., Kwon Y., Defretin Y. (2021). ultralytics/yolov5: V5.0-YOLOv5-P6 1280 models, AWS, Supervise.ly and YouTube integrations. Zenodo.

[17] Qiu S., Li Y., Zhao H., Li X., Yuan X. (2022). Foxtail Millet Ear Detection Method Based on Attention Mechanism and Improved YOLOv5. Sensors.

[18] Hou Q., Zhou D., Feng J. Coordinate Attention for Efficient Mobile Network Design. Proceedings of the IEEE/CVF Conference on Computer Vision and Pattern Recognition.

[19] Han K., Wang Y., Tian Q., Guo J., Xu C., Xu C. GhostNet: More Features From Cheap Operations. Proceedings of the IEEE/CVF Conference on Computer Vision and Pattern Recognition.

[20] Fan Y., Zhang S., Feng K., Qian K., Wang Y., Qin S. (2022). Strawberry Maturity Recognition Algorithm Combining Dark Channel Enhancement and YOLOv5. Sensors.

[21] Hameed Z., Wang C. Edge Detection Using Histogram Equalization and Multi-Filtering Process. Proceedings of the 2011 IEEE International Symposium on Circuits and Systems (ISCAS).

[22] Ng D., Chen Y., Tian B., Fu Q., Chng E. ConvMixer: Feature Interactive Convolution with Curriculum Learning for Small Footprint and Noisy Far-Field Keyword Spotting. Proceedings of the ICASSP 2022—2022 IEEE International Conference on Acoustics, Speech and Signal Processing (ICASSP).

[23] Wen G., Li S., Liu F., Luo X., Er M.J., Mahmud M., Wu T. (2023). YOLOv5s-CA: A Modified YOLOv5s Network with Coordinate Attention for Underwater Target Detection. Sensors.

[24] Hu J., Shen L., Sun G. (2018). Squeeze-and-Excitation Networks. Proceedings of the IEEE Conference on Computer Vision and Pattern Recognition (CVPR).

[25] Ogunrinde I., Bernadin S. A Review of the Impacts of Defogging on Deep Learning-Based Object Detectors in Self-Driving Cars. Proceedings of the SoutheastCon 2021.

[26] Zhang Z., Zhu W. (2024). YOLO-MFD: Remote sensing image object detection with multi-scale fusion dynamic head. Comput. Mater. Contin..

[27] Wang N., Wang Y., Feng Y., Wei Y. (2024). MDD-ShipNet: Math-Data Integrated Defogging for Fog-Occlusion Ship Detection. IEEE Trans. Intell. Transp. Syst..

[28] Zhang Y., Jia N. (2025). A target detection model HR-YOLO for advanced driver assistance systems in foggy conditions. Sci. Rep..

[29] Wang W., Jing B., Yu X., Zhang W., Wang S., Tang Z., Yang L. (2025). YOLO-Extreme: Obstacle Detection for Visually Impaired Navigation Under Foggy Weather. Sensors.

[30] Liu L., Zhang M., Zhai R., Zhou X., Wang D., Zhu S. (2025). Enhancing foggy day target detection using plant intelligence-inspired algorithms. Measurement.

[31] Li B., Peng X., Wang Z., Xu J., Feng D. AOD-Net: All-In-One Dehazing Network. Proceedings of the IEEE International Conference on Computer Vision (ICCV).

[32] Wang X., Girshick R., Gupta A., He K. (2018). Non-local Neural Networks. Proceedings of the IEEE/CVF Conference on Computer Vision and Pattern Recognition (CVPR).

[33] Wang Q., Wu B., Zhu P., Li P., Zuo W., Hu Q. (2020). ECA-Net: Efficient Channel Attention for Deep Convolutional Neural Networks. Proceedings of the IEEE/CVF Conference on Computer Vision and Pattern Recognition (CVPR).

[34] Ultralytics (2023). YOLOv8: Next-Generation Object Detection Model. https://github.com/ultralytics/ultralytics.

[35] Wang C.Y., Liao H.Y.M., Wu Y.H., Chen P.Y., Hsieh J.W., Yeh I.H. CSPNet: A New Backbone That Can Enhance Learning Capability of CNN. Proceedings of the IEEE/CVF Conference on Computer Vision and Pattern Recognition Workshops (CVPRW).

[36] Liu S., Qi L., Qin H., Shi J., Jia J. (2018). Path Aggregation Network for Instance Segmentation. Proceedings of the IEEE Conference on Computer Vision and Pattern Recognition (CVPR).

[37] Li X., Wang W., Wu L., Chen S., Hu X., Li J., Tang J., Yang J. Generalized Focal Loss: Learning Qualified and Distributed Bounding Boxes for Dense Object Detection. Proceedings of the Advances in Neural Information Processing Systems 33 (NeurIPS 2020).

[38] Zheng Z., Wang P., Liu W., Li J., Ye R., Ren D. (2020). Distance-IoU Loss: Faster and Better Learning for Bounding Box Regression. Proceedings of the AAAI Conference on Artificial Intelligence (AAAI).

[39] Mohan K., Sharma S. (2017). Recent Advances in Image Dehazing. IEEE J. Atmos. Sol. Terr. Phys..

[40] He K., Zhang X., Ren S., Sun J. (2016). Deep Residual Learning for Image Recognition. Proceedings of the IEEE Conference on Computer Vision and Pattern Recognition (CVPR).

[41] Roboflow Foggy PLXFM Dataset. https://universe.roboflow.com/foggy/foggy-plxfm.

